# (Benzyl­diphenyl­phosphane)chlorido­gold(I)

**DOI:** 10.1107/S1600536810045071

**Published:** 2010-11-10

**Authors:** Omar bin Shawkataly, Abu Tariq, Chin Sing Yeap, Hoong-Kun Fun

**Affiliations:** a Chemical Sciences Programme, School of Distance Education, Universiti Sains Malaysia, 11800 USM, Penang, Malaysia; b X-ray Crystallography Unit, School of Physics, Universiti Sains Malaysia, 11800 USM, Penang, Malaysia

## Abstract

In the title compound, [AuCl(C_19_H_17_P)], the Au^I^ atom exists within a P and Cl donor set that constitutes an almost linear geometry. The three phenyl rings make dihedral angles of 38.33 (14), 81.26 (15) and 81.28 (14)° with each other. In the crystal, mol­ecules are linked into chains along the *b* axis by inter­molecular C—H⋯Cl hydrogen bonds.

## Related literature

For general background to gold complexes, see: Parish & Cottrill (1987 ▶); Tiekink (2002 ▶); Baenziger *et al.* (1976 ▶); Chiu *et al.* (2009 ▶). For the synthesis of (CH_3_)_2_SAuCl, see: Francis (1901 ▶). For a related structure, see: Shawkataly *et al.* (2010 ▶). For the stability of the temperature controller used in the data collection, see: Cosier & Glazer (1986 ▶).
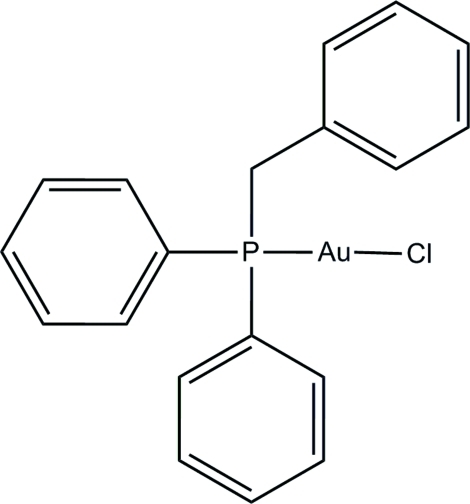


## Experimental

### 

#### Crystal data

[AuCl(C_19_H_17_P)]
*M* *_r_* = 508.71
Monoclinic, 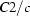

*a* = 16.1403 (11) Å
*b* = 9.0380 (7) Å
*c* = 23.5259 (17) Å
β = 91.012 (2)°
*V* = 3431.3 (4) Å^3^
*Z* = 8
Mo *K*α radiation
μ = 8.82 mm^−1^
*T* = 100 K
0.27 × 0.22 × 0.12 mm

#### Data collection

Bruker APEXII DUO CCD area-detector diffractometer
Absorption correction: multi-scan (*SADABS*; Bruker, 2009 ▶) *T*_min_ = 0.196, *T*_max_ = 0.408
28002 measured reflections
7489 independent reflections
6751 reflections with *I* > 2σ(*I*)
*R*_int_ = 0.030

#### Refinement

*R*[*F* ^2^ > 2σ(*F* ^2^)] = 0.028
*wR*(*F* ^2^) = 0.053
*S* = 1.14
7489 reflections
199 parameters
H-atom parameters constrained
Δρ_max_ = 1.43 e Å^−3^
Δρ_min_ = −2.38 e Å^−3^

### 

Data collection: *APEX2* (Bruker, 2009 ▶); cell refinement: *SAINT* (Bruker, 2009 ▶); data reduction: *SAINT*; program(s) used to solve structure: *SHELXTL* (Sheldrick, 2008 ▶); program(s) used to refine structure: *SHELXTL*; molecular graphics: *SHELXTL*; software used to prepare material for publication: *SHELXTL* and *PLATON* (Spek, 2009 ▶).

## Supplementary Material

Crystal structure: contains datablocks global, I. DOI: 10.1107/S1600536810045071/is2625sup1.cif

Structure factors: contains datablocks I. DOI: 10.1107/S1600536810045071/is2625Isup2.hkl

Additional supplementary materials:  crystallographic information; 3D view; checkCIF report

## Figures and Tables

**Table:** 

Au1—P1	2.2292 (7)
Au1—Cl1	2.2983 (7)

**Table:** 

P1—Au1—Cl1	173.62 (2)

**Table 2:** Hydrogen-bond geometry (Å, °)

*D*—H⋯*A*	*D*—H	H⋯*A*	*D*⋯*A*	*D*—H⋯*A*
C7—H7*B*⋯Cl1^i^	0.97	2.71	3.675 (3)	175

Symmetry code: (i) 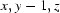.

**Fig. 1.**
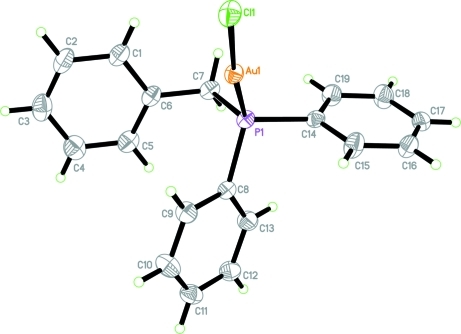
The molecular structure of the title compound with 50% probability ellipsoids for non-H atoms.

**Fig. 2.**
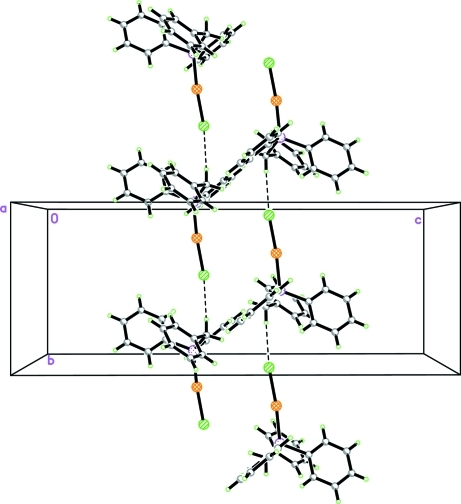
The crystal packing of the title compound, viewed down the a axis, showing the molecules linked into chains along the b axis. Intermolecular hydrogen bonds are shown as dashed lines.

## supplementary crystallographic information

### Comment

Gold and gold compounds have been used for medicinal purposes over a long period of time (Parish and Cottrill, 1987). Phosphinegold (I) forms an important class of compounds of gold (Baenziger *et al.*, 1976). Their thiolate derivatives are compounds with well known medicinal properties (Tiekink, 2002). They are conveniently prepared from their phosphinegold(I) chloride precursors and it is in this context that the title compound C_6_H_5_CH_2_P(C_6_H_5_)_2_AuCl, was prepared and characterized. Complex of Iridium (III) with benzyldiphenyl phospine is reported (Chiu *et al.*, 2009), however, no such metal complex with gold (I) is known. Herein, we report the crystal structure of the title complex C_6_H_5_CH_2_P(C_6_H_5_)_2_AuCl.

In the title compound (Fig. 1), the P1–Au1–Cl1 is almost linear with an angle of 173.62 (2)°. The three phosphine-substituted phenyl rings (C1–C6, C8–C13 and C14–C19) make dihedral angles of 38.33 (14), 81.26 (15) and 81.28 (14)° with each other (C1–C6/C8–C13, C1–C6/C14–C19 and C8–C13/C14–C19). The geometric parameters are comparable to its related structure (Shawkataly *et al.*, 2010). In the crystal structure, the molecules are linked into chains along the *b* axis by intermolecular C7—H7B···Cl1 hydrogen bonds (Fig. 2, Table 2).

### Experimental

The title compound was prepared by mixing equimolar quantities of Me_2_SAuCl, obtained as per conventional method (Francis, 1901) and C_6_H_5_CH_2_P(C_6_H_5_)_2_ (Strem Chemicals Co. Ltd.) in CH_2_Cl_2_ held at room temperature. The solution was stirred for 2 h and white crystalline solid was recovered after the removal of solvent under vacuum. The colourless plate-like crystals were obtained in 90% yield from the layering of methanol over a concentrated dichloromethane solution of the compound (m.p. 208 °C, decomposition) kept at refrigerator for couple of days.

### Refinement

All hydrogen atoms were positioned geometrically (C—H = 0.93 or 0.97 Å) and refined using a riding model, with *U*_iso_(H) = 1.2*U*_eq_(C). The maximum and minimum residual electron density peaks of 1.43 and -2.38 e Å^-3^, respectively, were located 0.73 and 0.60 Å from the Au1 and Cl1 atom, respectively.

### Crystal data

**Table:**

[AuCl(C_19_H_17_P)]	*F*(000) = 1936
*M**_r_* = 508.71	*D*_x_ = 1.969 Mg m^−^^3^
Monoclinic, *C*2/*c*	Mo *K*α radiation, λ = 0.71073 Å
Hall symbol: -C 2yc	Cell parameters from 9893 reflections
*a* = 16.1403 (11) Å	θ = 2.7–37.4°
*b* = 9.0380 (7) Å	µ = 8.82 mm^−^^1^
*c* = 23.5259 (17) Å	*T* = 100 K
β = 91.012 (2)°	Plate, colourless
*V* = 3431.3 (4) Å^3^	0.27 × 0.22 × 0.12 mm
*Z* = 8	

### Data collection

**Table:**

Bruker APEXII DUO CCD area-detector diffractometer	7489 independent reflections
Radiation source: fine-focus sealed tube	6751 reflections with *I* > 2σ(*I*)
graphite	*R*_int_ = 0.030
φ and ω scans	θ_max_ = 35.0°, θ_min_ = 2.7°
Absorption correction: multi-scan (*SADABS*; Bruker, 2009)	*h* = −26→26
*T*_min_ = 0.196, *T*_max_ = 0.408	*k* = −7→14
28002 measured reflections	*l* = −37→37

### Refinement

**Table:**

Refinement on *F*^2^	Primary atom site location: structure-invariant direct methods
Least-squares matrix: full	Secondary atom site location: difference Fourier map
*R*[*F*^2^ > 2σ(*F*^2^)] = 0.028	Hydrogen site location: inferred from neighbouring sites
*wR*(*F*^2^) = 0.053	H-atom parameters constrained
*S* = 1.14	*w* = 1/[σ^2^(*F*_o_^2^) + (0.*P*)^2^ + 12.0783*P*] where *P* = (*F*_o_^2^ + 2*F*_c_^2^)/3
7489 reflections	(Δ/σ)_max_ = 0.002
199 parameters	Δρ_max_ = 1.43 e Å^−^^3^
0 restraints	Δρ_min_ = −2.37 e Å^−^^3^

### Special details

**Table:**

Experimental. The crystal was placed in the cold stream of an Oxford Cryosystems Cobra open-flow nitrogen cryostat (Cosier & Glazer, 1986) operating at 100.0 (1) K.
Geometry. All e.s.d.'s (except the e.s.d. in the dihedral angle between two l.s. planes) are estimated using the full covariance matrix. The cell e.s.d.'s are taken into account individually in the estimation of e.s.d.'s in distances, angles and torsion angles; correlations between e.s.d.'s in cell parameters are only used when they are defined by crystal symmetry. An approximate (isotropic) treatment of cell e.s.d.'s is used for estimating e.s.d.'s involving l.s. planes.
Refinement. Refinement of *F*^2^ against ALL reflections. The weighted *R*-factor *wR* and goodness of fit *S* are based on *F*^2^, conventional *R*-factors *R* are based on *F*, with *F* set to zero for negative *F*^2^. The threshold expression of *F*^2^ > σ(*F*^2^) is used only for calculating *R*-factors(gt) *etc*. and is not relevant to the choice of reflections for refinement. *R*-factors based on *F*^2^ are statistically about twice as large as those based on *F*, and *R*- factors based on ALL data will be even larger.

### Fractional atomic coordinates and isotropic or equivalent isotropic displacement parameters (Å^2^)

**Table:**

	*x*	*y*	*z*	*U*_iso_*/*U*_eq_	
Au1	0.323189 (5)	0.699677 (10)	0.101304 (4)	0.01995 (3)	
Cl1	0.31261 (4)	0.94885 (7)	0.08307 (3)	0.02787 (12)	
P1	0.34216 (4)	0.45646 (7)	0.11042 (3)	0.01953 (11)	
C1	0.11935 (17)	0.4585 (3)	0.08496 (12)	0.0293 (5)	
H1A	0.1301	0.5168	0.0534	0.035*	
C2	0.04361 (18)	0.4704 (4)	0.11189 (14)	0.0354 (6)	
H2A	0.0039	0.5364	0.0980	0.042*	
C3	0.02680 (18)	0.3854 (4)	0.15894 (14)	0.0367 (7)	
H3A	−0.0241	0.3937	0.1766	0.044*	
C4	0.08609 (19)	0.2873 (4)	0.17987 (14)	0.0353 (6)	
H4A	0.0754	0.2306	0.2119	0.042*	
C5	0.16161 (18)	0.2743 (3)	0.15271 (13)	0.0304 (5)	
H5A	0.2010	0.2077	0.1665	0.036*	
C6	0.17911 (15)	0.3596 (3)	0.10519 (11)	0.0243 (5)	
C7	0.26100 (16)	0.3445 (3)	0.07589 (11)	0.0238 (4)	
H7A	0.2545	0.3751	0.0365	0.029*	
H7B	0.2777	0.2414	0.0762	0.029*	
C8	0.34892 (14)	0.3852 (3)	0.18224 (10)	0.0210 (4)	
C9	0.31074 (17)	0.4608 (3)	0.22617 (11)	0.0264 (5)	
H9A	0.2867	0.5528	0.2195	0.032*	
C10	0.3088 (2)	0.3977 (4)	0.27999 (12)	0.0334 (6)	
H10A	0.2819	0.4465	0.3092	0.040*	
C11	0.34654 (19)	0.2628 (4)	0.29046 (12)	0.0305 (5)	
H11A	0.3461	0.2224	0.3268	0.037*	
C12	0.38490 (18)	0.1881 (3)	0.24694 (13)	0.0313 (5)	
H12A	0.4097	0.0969	0.2540	0.038*	
C13	0.38647 (18)	0.2485 (3)	0.19300 (12)	0.0284 (5)	
H13A	0.4125	0.1981	0.1638	0.034*	
C14	0.43910 (15)	0.4021 (3)	0.07884 (10)	0.0208 (4)	
C15	0.51087 (17)	0.4641 (4)	0.10215 (13)	0.0322 (6)	
H15A	0.5074	0.5293	0.1326	0.039*	
C16	0.58762 (17)	0.4292 (4)	0.08027 (13)	0.0305 (5)	
H16A	0.6355	0.4708	0.0960	0.037*	
C17	0.59297 (17)	0.3323 (3)	0.03495 (12)	0.0277 (5)	
H17A	0.6445	0.3080	0.0205	0.033*	
C18	0.52207 (19)	0.2721 (4)	0.01125 (13)	0.0349 (6)	
H18A	0.5259	0.2081	−0.0196	0.042*	
C19	0.44462 (17)	0.3060 (3)	0.03307 (12)	0.0296 (5)	
H19A	0.3969	0.2645	0.0170	0.036*	

### Atomic displacement parameters (Å^2^)

**Table:**

	*U*^11^	*U*^22^	*U*^33^	*U*^12^	*U*^13^	*U*^23^
Au1	0.01950 (4)	0.01675 (4)	0.02361 (4)	0.00189 (3)	0.00046 (3)	−0.00088 (3)
Cl1	0.0251 (3)	0.0164 (2)	0.0422 (3)	0.0030 (2)	0.0035 (2)	0.0030 (2)
P1	0.0198 (2)	0.0172 (3)	0.0215 (3)	0.0010 (2)	−0.0013 (2)	−0.0018 (2)
C1	0.0257 (11)	0.0302 (13)	0.0317 (13)	0.0055 (10)	−0.0051 (10)	−0.0069 (10)
C2	0.0240 (12)	0.0407 (17)	0.0414 (16)	0.0082 (11)	−0.0059 (11)	−0.0109 (13)
C3	0.0254 (12)	0.0434 (18)	0.0412 (16)	−0.0034 (12)	0.0006 (11)	−0.0158 (13)
C4	0.0312 (13)	0.0360 (16)	0.0387 (15)	−0.0118 (12)	0.0010 (11)	−0.0027 (12)
C5	0.0278 (12)	0.0252 (13)	0.0381 (14)	−0.0022 (10)	−0.0032 (10)	0.0006 (10)
C6	0.0218 (10)	0.0208 (11)	0.0302 (12)	−0.0006 (8)	−0.0048 (9)	−0.0058 (9)
C7	0.0251 (10)	0.0199 (10)	0.0262 (11)	0.0007 (9)	−0.0040 (9)	−0.0037 (8)
C8	0.0201 (9)	0.0202 (10)	0.0228 (10)	0.0004 (8)	−0.0015 (8)	0.0001 (8)
C9	0.0300 (12)	0.0212 (11)	0.0279 (12)	0.0021 (9)	0.0018 (9)	−0.0007 (9)
C10	0.0443 (16)	0.0291 (13)	0.0270 (12)	−0.0017 (12)	0.0070 (11)	−0.0016 (10)
C11	0.0350 (13)	0.0309 (14)	0.0255 (12)	−0.0053 (11)	0.0000 (10)	0.0042 (10)
C12	0.0322 (13)	0.0298 (14)	0.0319 (13)	0.0053 (11)	0.0004 (10)	0.0087 (11)
C13	0.0309 (12)	0.0258 (12)	0.0287 (12)	0.0081 (10)	0.0022 (10)	0.0035 (10)
C14	0.0217 (9)	0.0188 (10)	0.0219 (10)	0.0041 (8)	−0.0011 (8)	0.0012 (8)
C15	0.0241 (11)	0.0358 (15)	0.0367 (14)	0.0007 (11)	−0.0008 (10)	−0.0128 (12)
C16	0.0221 (11)	0.0350 (15)	0.0343 (13)	0.0024 (10)	−0.0021 (10)	−0.0026 (11)
C17	0.0251 (11)	0.0327 (14)	0.0253 (11)	0.0041 (10)	0.0036 (9)	0.0037 (10)
C18	0.0314 (13)	0.0429 (18)	0.0305 (13)	0.0014 (12)	0.0055 (11)	−0.0118 (12)
C19	0.0271 (11)	0.0351 (14)	0.0266 (12)	0.0002 (11)	0.0002 (9)	−0.0081 (11)

### Geometric parameters (Å, °)

**Table:**

Au1—P1	2.2292 (7)	C9—C10	1.389 (4)
Au1—Cl1	2.2983 (7)	C9—H9A	0.9300
P1—C8	1.810 (3)	C10—C11	1.383 (5)
P1—C14	1.812 (2)	C10—H10A	0.9300
P1—C7	1.834 (3)	C11—C12	1.382 (4)
C1—C2	1.391 (4)	C11—H11A	0.9300
C1—C6	1.393 (4)	C12—C13	1.382 (4)
C1—H1A	0.9300	C12—H12A	0.9300
C2—C3	1.379 (5)	C13—H13A	0.9300
C2—H2A	0.9300	C14—C19	1.387 (4)
C3—C4	1.388 (5)	C14—C15	1.391 (4)
C3—H3A	0.9300	C15—C16	1.386 (4)
C4—C5	1.391 (4)	C15—H15A	0.9300
C4—H4A	0.9300	C16—C17	1.383 (4)
C5—C6	1.391 (4)	C16—H16A	0.9300
C5—H5A	0.9300	C17—C18	1.376 (4)
C6—C7	1.508 (4)	C17—H17A	0.9300
C7—H7A	0.9700	C18—C19	1.394 (4)
C7—H7B	0.9700	C18—H18A	0.9300
C8—C9	1.391 (4)	C19—H19A	0.9300
C8—C13	1.398 (4)		
			
P1—Au1—Cl1	173.62 (2)	C10—C9—C8	119.6 (3)
C8—P1—C14	104.37 (11)	C10—C9—H9A	120.2
C8—P1—C7	104.37 (12)	C8—C9—H9A	120.2
C14—P1—C7	106.50 (12)	C11—C10—C9	120.4 (3)
C8—P1—Au1	116.52 (8)	C11—C10—H10A	119.8
C14—P1—Au1	110.26 (8)	C9—C10—H10A	119.8
C7—P1—Au1	113.94 (9)	C12—C11—C10	120.0 (3)
C2—C1—C6	120.2 (3)	C12—C11—H11A	120.0
C2—C1—H1A	119.9	C10—C11—H11A	120.0
C6—C1—H1A	119.9	C13—C12—C11	120.2 (3)
C3—C2—C1	120.6 (3)	C13—C12—H12A	119.9
C3—C2—H2A	119.7	C11—C12—H12A	119.9
C1—C2—H2A	119.7	C12—C13—C8	120.0 (3)
C2—C3—C4	119.8 (3)	C12—C13—H13A	120.0
C2—C3—H3A	120.1	C8—C13—H13A	120.0
C4—C3—H3A	120.1	C19—C14—C15	119.6 (2)
C3—C4—C5	119.7 (3)	C19—C14—P1	123.8 (2)
C3—C4—H4A	120.2	C15—C14—P1	116.63 (19)
C5—C4—H4A	120.2	C16—C15—C14	120.4 (3)
C6—C5—C4	120.9 (3)	C16—C15—H15A	119.8
C6—C5—H5A	119.5	C14—C15—H15A	119.8
C4—C5—H5A	119.5	C17—C16—C15	119.9 (3)
C5—C6—C1	118.8 (3)	C17—C16—H16A	120.0
C5—C6—C7	120.6 (2)	C15—C16—H16A	120.0
C1—C6—C7	120.6 (3)	C18—C17—C16	120.0 (3)
C6—C7—P1	111.89 (17)	C18—C17—H17A	120.0
C6—C7—H7A	109.2	C16—C17—H17A	120.0
P1—C7—H7A	109.2	C17—C18—C19	120.6 (3)
C6—C7—H7B	109.2	C17—C18—H18A	119.7
P1—C7—H7B	109.2	C19—C18—H18A	119.7
H7A—C7—H7B	107.9	C14—C19—C18	119.6 (3)
C9—C8—C13	119.7 (2)	C14—C19—H19A	120.2
C9—C8—P1	119.93 (19)	C18—C19—H19A	120.2
C13—C8—P1	120.16 (19)		
			
C6—C1—C2—C3	0.3 (5)	C8—C9—C10—C11	1.9 (5)
C1—C2—C3—C4	0.3 (5)	C9—C10—C11—C12	−1.5 (5)
C2—C3—C4—C5	−0.8 (5)	C10—C11—C12—C13	0.7 (5)
C3—C4—C5—C6	0.8 (5)	C11—C12—C13—C8	−0.3 (5)
C4—C5—C6—C1	−0.2 (4)	C9—C8—C13—C12	0.6 (4)
C4—C5—C6—C7	−179.8 (3)	P1—C8—C13—C12	−174.1 (2)
C2—C1—C6—C5	−0.4 (4)	C8—P1—C14—C19	116.6 (2)
C2—C1—C6—C7	179.3 (3)	C7—P1—C14—C19	6.5 (3)
C5—C6—C7—P1	−83.8 (3)	Au1—P1—C14—C19	−117.6 (2)
C1—C6—C7—P1	96.6 (3)	C8—P1—C14—C15	−64.5 (2)
C8—P1—C7—C6	60.1 (2)	C7—P1—C14—C15	−174.6 (2)
C14—P1—C7—C6	170.15 (18)	Au1—P1—C14—C15	61.3 (2)
Au1—P1—C7—C6	−68.1 (2)	C19—C14—C15—C16	−0.6 (5)
C14—P1—C8—C9	147.9 (2)	P1—C14—C15—C16	−179.6 (2)
C7—P1—C8—C9	−100.5 (2)	C14—C15—C16—C17	0.1 (5)
Au1—P1—C8—C9	26.0 (2)	C15—C16—C17—C18	0.7 (5)
C14—P1—C8—C13	−37.4 (2)	C16—C17—C18—C19	−0.9 (5)
C7—P1—C8—C13	74.2 (2)	C15—C14—C19—C18	0.4 (4)
Au1—P1—C8—C13	−159.25 (19)	P1—C14—C19—C18	179.2 (2)
C13—C8—C9—C10	−1.4 (4)	C17—C18—C19—C14	0.4 (5)
P1—C8—C9—C10	173.3 (2)		

### Hydrogen-bond geometry (Å, °)

**Table:**

*D*—H···*A*	*D*—H	H···*A*	*D*···*A*	*D*—H···*A*
C7—H7B···Cl1^i^	0.97	2.71	3.675 (3)	175

Symmetry codes: (i) *x*, *y*−1, *z*.
